# Enhancing genomic‐based forward prediction accuracy in wheat by integrating UAV‐derived hyperspectral and environmental data with machine learning under heat‐stressed environments

**DOI:** 10.1002/tpg2.20554

**Published:** 2025-01-08

**Authors:** Jordan McBreen, Md Ali Babar, Diego Jarquin, Yiannis Ampatzidis, Naeem Khan, Sudip Kunwar, Janam Prabhat Acharya, Samuel Adewale, Gina Brown‐Guedira

**Affiliations:** ^1^ Department of Agronomy University of Florida Gainesville Florida USA; ^2^ Agricultural and Biological Engineering Department, Southwest Florida Research and Education Center University of Florida, IFAS Immokalee Florida USA; ^3^ USDA‐ARS Southeast Area, Plant Science Research Raleigh North Carolina USA

## Abstract

Integrating genomic, hyperspectral imaging (HSI), and environmental data enhances wheat yield predictions, with HSI providing detailed spectral insights for predicting complex grain yield (GY) traits. Incorporating HSI data with single nucleotide polymorphic markers (SNPs) resulted in a substantial improvement in predictive ability compared to the conventional genomic prediction models. Over the course of several years, the prediction ability varied due to diverse weather conditions. The most comprehensive parametric model tested, which included SNPs, HSI, and environmental covariates data, consistently achieved the best results, closely followed by machine learning (ML) approaches when considering the same omics data. For example, the most comprehensive model (M9), under the forward prediction cross‐validation scheme, predicted the GY of the 2023 growing season using data from 2021 and 2022 for a correlation between predicted and observed values of 0.53. This model demonstrated superior performance compared to less complex models, emphasizing the advantage of integrating numerous data sources and their interactive effects. Furthermore, when comparing the top 25% of the predicted lines versus the corresponding observed lines with the highest GY, the M9 model returned a coincide index (CI) of 55% (i.e., in both sets, 55% of the top 25% values were common), whereas for the highest performing ML model (gradient boosting regression), the CI was of 46%. This study highlights the potential of multi‐data source approaches to accelerate the selection of heat‐tolerant wheat genotypes.

## Core Ideas

- Aerial hyperspectral imaging (HSI) and environmental covariates (ECs) improve wheat yield predictions.
- Combining single nucleotide polymorphisms, HSI, and ECs enhances prediction accuracy, especially for forward predictions across environments.
- Machine learning models like gradient boosting regression and random forest regression rival genomic models when using diverse and integrated data sources.
- Multi‐omics data integration improves genomic models for selecting heat‐tolerant and high‐yielding wheat lines.

**Abbreviations**
ANN: artificial neural network
BLUE: best linear unbiased estimate
CV: cross‐validation
EC: environmental covariate
G × E: genotype by environment interaction
GBR: gradient boosting regression
GY: grain yield
HSI: hyperspectral imaging
RBF: radial basis function
RFR: random forest regression
RKHS: reproducing kernel Hilbert space
SNP: single nucleotide polymorphism
SVMR: support vector machine regression
UAV: uncrewed aerial vehicle

## 1 INTRODUCTION

*Triticum aestivum*, more commonly referred to as wheat, is a vital food crop in global agriculture, accounting for more than 95% of total wheat production around the world and serving some 30% of the world's population as a primary source of daily nutritionary value (Dohlman et al., 2022; Giraldo et al., 2019). Wheat is cultivated on over 218 million ha of land globally and has been known to achieve average yields of 3300 kg ha^−1^ (Erenstein et al., 2022). However, abiotic environmental constraints such as drought and high‐temperature stress pose significant threats to wheat production. Accounting for climatic trends, there has been reported a decline of ∼6% in wheat output around the globe since 1980 due to steadily rising temperatures and increasing unpredictability in weather events (Zhu et al., 2023). Unusually high temperatures can be particularly harmful during the reproductive stages in wheat, causing the abortion of florets, accelerated maturity, and therefore reduced grain‐filling periods, and overall reduced grain weight and number (Farooq et al., 2015; Raza et al., 2023). Current climate projection models have suggested global temperature increases of up to 2°C by the year 2050, which could potentially exacerbate yield losses (Zencirci, 2024). Thus, to ensure the food security of the current projected global population of 10 billion people by 2050, wheat production must increase by 60% compared to the actual levels. It is equivalent to a sustainable annual increase of more than 1.6% per year (Mittal, 2022; H. Sun et al., 2023). This outlines the absolute necessity for developing hardy new wheat varieties that maintain high GY under diverse environmental conditions while demonstrating resilience to sudden changes in climate patterns. Despite wheat's elasticity to adapt to and be grown in various climates while maintaining high standards of quality traits, it still must overcome limitations on GY that can be due to unfavorable environmental conditions, subpar genetics, and interactions of the two factors. Current global genetic gains in wheat yield are estimated at under 1% annually (Krishnappa et al., 2022). This highlights an urgent need for innovative breeding approaches that integrate accurate phenotyping and the prediction of complex traits. By improving performance across different environments and breeding lines, these strategies will help meet the growing global demand for wheat (Ahmad et al., 2023). Given the increasing temperatures and growingly unpredictable rainfall, breeders have largely begun to prioritize both production potential and yield stability in wheat, for deriving robust varieties equipped with the ability to sustain acceptable yields under abiotic stressors such as high temperatures (Tyagi & Pandey, 2022). High temperatures accelerate crucial growth phases, thereby reducing the period allotted for carbon absorption and assimilation, hence decreasing spike fertility during flowering (Al‐Khatib & Paulsen, 1984; Langridge & Reynolds, 2021).

Complex quantitative traits such as GY are influenced by many genes with small and, in some cases, almost imperceptible effects. Therefore, leveraging most, if not all, available genomic marker information can be important to develop accurate prediction models (Bassi et al., 2016; Meuwissen et al., 2022). Genomic prediction (GP) uses single nucleotide polymorphism (SNP) markers scattered across the genome to predict the genomic estimated breeding values of genotypes even before they are phenotyped. Under the traditional GP framework, a training population consisting of both phenotypic and genomic data is used to build a predictive model, often leveraging genetic relatedness or marker effects, which is then used to predict trait performance in a separate validation population (VP). Before implementing GP in real life scenarios, it is necessary to evaluate the potential of the available data to deliver accurate prediction values of the unobserved genotypes. For this, a cross‐validation (CV) study is conducted where the VP consists of lines for which certain genotyping or phenotyping data are intentionally masked as missing to simulate unobserved conditions. The predictive ability of the models is then measured as the Pearson correlation between predicted and observed values (Guo, Khan, et al., 2020). GP has been shown to be superior to more parsimonious strategies such as marker‐assisted selection (MAS) methods because it leverages a litany of markers with various effect sizes rather than relying on just a few key markers (Bernardo, 2020).

The application of high throughput phenotyping (HTP) has enhanced GP by integrating diverse data from different traits that are known to show correlations with yield and thereby improve model's predictive ability. HTP also allows breeders to better quantify the genotype‐by‐environment interaction (G × E) effects. This can be achieved by combining different data sources, including omics data such as genomics, transcriptomics, or metabolomics, along with environmental variables like precipitation, temperature, and soil conditions. G × E can be understood as the variations in genotypic rankings across growing environments or years, with stable genotypes maintaining relative performance and yield across multiple conditions (Dwivedi et al., 2020). Many field‐based platforms, such as ground‐based mobile systems and uncrewed aerial vehicles (UAVs), have streamlined data collection, making HTP collection amenable to breeders of all calibers. Much of the breeding research that uses UAV‐based HTP has gravitated toward the use of poly‐copters over fixed‐wing UAVs because, despite often being able to carry a smaller payload, they allow for greater mobility, fly at a large range of altitudes, and maintain a stationary position in the air if needed (Mishra, 2021; Shi et al., 2016). They also allow the easy collection of time‐series data over the most crucial portions of the wheat growth cycle, which could offer breeders the ability to make early‐stage selections in preceding years (J. Sun et al., 2019).

Hyperspectral imaging (HSI) holds the potential to capture very detailed spectral information by using hundreds of narrowband channels from across the spectral range of ∼400–2500 µm (Signoroni et al., 2019). Because HSI quickly measures the reflection properties of different materials and instantly reports their spectral signatures, it is a valuable resource for phenotyping wheat varieties. Hence, HSI offers the potential to assist in the selection of genotypes that have not largely been fully explored. Similar to SNP markers, it has been found that incorporating all HSI bands into prediction models for GY outperforms those models that only use subsets of bands or vegetation indices (VIs) derived from RGB or multispectral imaging (Montesinos‐López et al., 2017; Persa et al., 2021). Hence, aerial HSI shows great potential to serve as a powerful tool for physiological selection and GP coupled with HTP, offering the ability to sample high‐level information on numerous traits simultaneously and rapidly. In the framework of multivariate GP modeling, which integrates multiple data sources or omics in the prediction process, HSI integration has been shown to deliver superior accuracy compared to univariate models (Bhatta et al., 2020; Krause et al., 2019; Michel et al., 2019). These multivariate models can use correlations among several different predictor variables like HSI data, genetic markers, and information about the growing environment in the form of environmental covariates (ECs) to enhance predictions. Such models can provide a more precise approach because they often better account for the complex interactions between these factors within the model's TP. The utilization of data from multiple sources collected via HTP methods has shown great potential for increasing GP efficiency (Guo, Khan, et al., 2020).

**Core Ideas**

- Aerial hyperspectral imaging (HSI) and environmental covariates (ECs) improve wheat yield predictions.
- Combining single nucleotide polymorphisms, HSI, and ECs enhances prediction accuracy, especially for forward predictions across environments.
- Machine learning models like gradient boosting regression and random forest regression rival genomic models when using diverse and integrated data sources.
- Multi‐omics data integration improves genomic models for selecting heat‐tolerant and high‐yielding wheat lines.

Along with spectral and other forms of locally collected HTP data are ECs such as temperature, rainfall, and daily solar radiation that can be attained from online publicly available weather databases. These covariates can help characterize a growing environment more accurately by incorporating differences in weather patterns from 1 year to the next or from one environment to another, allowing the models to even further characterize the G × E component improving the model's stability over different growing environments (Wang et al., 2018). Modeling G × E is essential for building GP models that remain relevant year after year.

Effective predictive models rely heavily on the quality and size of the training dataset to accurately capture the relationships between genotype, phenotype, and environment (Spindel et al., 2016). Studies in recent years have explored various approaches for GP of grain yield (GY) and related traits, comparing their effectiveness in diverse scenarios (Kaushal et al., 2024; Ma et al., 2018; Rachmatia et al., 2017). Limited or incomplete data, such as small population sizes or missing markers, can restrict performance, with a sufficiently large and high‐quality dataset being an important prerequisite in these modeling approaches (Domingos, 2012). Integrating diverse data sources, such as HSI and ECs, has been shown to enhance predictions by providing additional layers of information, particularly for improving predictions of complex traits in challenging scenarios (Costa et al., 2021).

The current research aims to integrate aerial HTP, ECs, and genomic marker data with phenotypic records to improve genomic predictive ability in a wheat breeding program under heat‐stressed environmental conditions across years. The development and testing of multivariate, as well as ML‐based prediction models incorporating diverse data types, was accomplished to enhance predictions made on un‐phenotyped lines for improving the selections of the top year‐to‐year performing wheat lines. The significance of this research lies in its capacity to integrate multiple areas of expertise regarding the implementation of aerially derived HTP data and high‐level modeling approaches in GP for wheat breeding. Assimilating both conventional and ML‐based prediction modeling with multiple HTP data types/omics to better understand how these facets of modern yield predictive modeling can work synergistically to produce robust and repeatable models with high predictive performance.

## 2 MATERIALS AND METHODS

### 2.1 Experimental design, environment, and genetic material used

**FIGURE 1:**
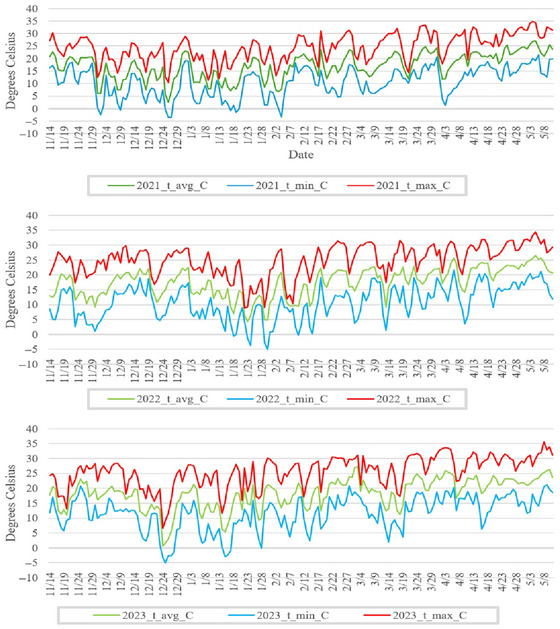
Growing season temperatures in Citra, FL. Temperature graph in degrees Celsius showing daily maximum (red), average (green), and minimum (blue) temperatures during the specified period for three consecutive years. The data span from November to May for the years 2021 (top chart), 2022 (middle chart), and 2023 (bottom chart).

Field‐based data collection for research was carried out during the growing seasons of three consecutive years (2021–2023) at the University of Florida Plant Science and Research Education Unit (PSREU) in Citra, FL. The plants used in this study are soft red winter wheat lines bred to be adapted to the environments of the southeastern United States and are known to perform reasonably well under these environmental conditions. They are derived from the SunGrains cooperative breeding initiative and contain a mixture of lines from the University of Arkansas, Clemson University, University of Florida, University of Georgia, Louisiana State University, North Carolina State University, and Texas A&M University. The number of wheat lines grown and had data collected throughout the 3 years in which the experiment was conducted were 330 genotypes in 2021, 262 in 2022, and 139 in 2023. A total of 48 lines were shared from 2021 to 2022, 50 were shared between 2022 and 2023, 12 lines were shared between 2021 and 2023, and six lines were common across all 3 years. Based on this, it is apparent that not all lines remain common across each year but that some lines are consistent from year to year, suggestive of the unbalanced nature of this type of dataset. The field trials were conducted using an alpha lattice design with two replicates for each genotype in each year. This design was chosen to account for spatial variation within the field and improve the precision of the phenotypic measurements. All lines for which data were collected were grown in plots measuring 5.3 m^2^. During the post‐anthesis/grain‐filling stages of wheat growth in Citra, FL, the temperatures can reach upward of 30°C (Figure 1), which is generally higher than in most other wheat growing locations in the United States. This would indicate that the ambient temperature in Citra during the wheat growing months (November–May) is ideal for identifying heat‐tolerant wheat genotypes and developing physiological indicator traits. Due to the occurrence of high temperatures during the crucial physiological stages of their growth cycle, the wheat genotypes in this experiment can be classified as heat‐stressed during the post‐flowering portion of their lifecycle.

### 2.2 Traits measurement

Records were collected for days to heading (DTH), GY, ECs, and HSI data. The DTH value measured for a plot is the number of days that have passed since the initial date of planting, at which at least 50% of the plants within the plot have headed based on the Zadoks growth scale and is used to control for the different rates at which the genotypes mature. Grain weight data were collected with a single plot combine harvester to harvest all rows. After harvest, GY is calculated by dividing the total weight of grain by the area of the plot at a moisture content of 13%.

Environmental covariate data were collected from the Florida Automated Weather Network database, which is available to the public. The ECs dataset is an extensive collection of environmental variables that were taken over the growth seasons of 2021–2023 with the purpose of characterizing the impact of these covariates on different wheat genotypes throughout the years. The environmental dataset consists of daily values for each ECs that have been grouped into 10‐day intervals, where the mean values are calculated by averaging the daily observations within each interval. Covering the period from November 11 to May 11 for each growing season, the included key environmental characteristics are the average temperature (t_avg_C), lowest temperature (t_min_C), maximum temperature (t_max_C), relative humidity (rel_hum), total rainfall (rain_cm), and solar radiation (solrad_W/m^2^). Here, W/m^2^ (watts per square meter) quantifies the power of solar energy received per unit area, a metric important for better understanding the energy available for processes like photosynthesis. The documentation of each characteristic across several 10‐day periods offers a chronological perspective on the prevailing growth conditions in Citra, FL, for each year. This dataset enables the examination of G × E by establishing a correlation between environmental variables and a genotype's performance over time, while also allowing for monitoring of the timing and impact of environmental factors on plant development and the analysis of more complex temporal patterns. Aggregated ECs data for each month within each year are displayed in Table 1.

**TABLE 1:** Monthly averages of environmental covariates (ECs) in Citra, FL (2021–2023). Monthly averages for the environmental covariates (ECs) of relative humidity (%), rainfall (cm), and solar radiation (W/m^2^) from November to May for the years of 2021–2023 in Citra, FL.

Month	Relative humidity (%) 2021	Relative humidity (%) 2022	Relative humidity (%) 2023	Rainfall (cm) 2021	Rainfall (cm) 2022	Rainfall (cm) 2023	Solar radiation (W/m^2^) 2021	Solar radiation (W/m^2^) 2022	Solar radiation (W/m^2^) 2023
Nov	83.5	79.7	82.3	0.38	0.15	0.30	119.2	126.0	89.8
Dec	81.8	90.0	83.8	0.81	1.49	0.43	107.6	96.3	85.28
Jan.	80.1	78.3	78.3	0.36	1.02	0.98	113.1	129.0	125.7
Feb.	80.6	79.2	79.6	3.73	0.86	0.89	137.9	143.9	150.98
March	79.0	80.1	70.1	1.68	13.5	2.15	176.2	146.6	241.68
April	74.5	75.5	72.5	6.55	4.01	1.85	210.1	218.9	220.0
May	77.2	76.8	71.9	4.59	2.34	2.81	208.2	253.1	273.8

### 2.3 Aerial high throughput phenotyping (HTP)

**FIGURE 2:**
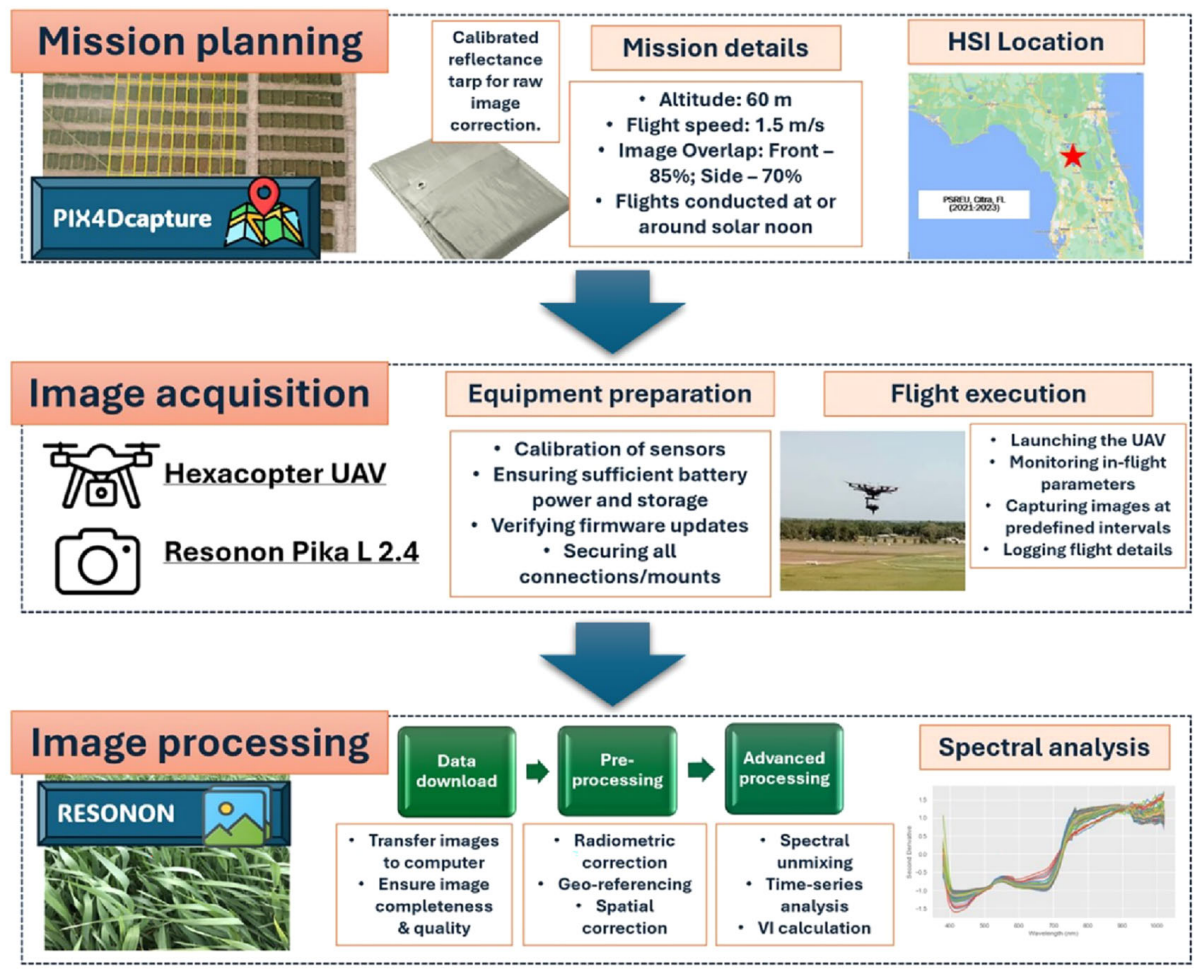
Hyperspectral data collection workflow. Workflow diagram outlining the steps for hyperspectral imaging (HSI) data collection and processing divided into three main stages: mission planning, image acquisition, and image processing. Specific tasks, including the planning of flight missions, the preparation of equipment, and the application of advanced image processing techniques, are associated with each stage. This methodology guarantees the systematic collection, processing, and analysis of high‐quality hyperspectral data to produce reliable and accurate results. UAV, uncrewed aerial vehicle; VI, vegetation index.

The aerial HSI data were acquired each year via a six‐propeller hexacopter UAV with a diagonal wheelbase size of 1133 mm and a weight of ∼9.5 kg with batteries included. The HTP data collection device used to capture the inflight data was the Resonon Pika L 2.4 (Resonon Inc.). The UAV‐based imaging system includes: (1) Resonon Pika L 2.4 hyperspectral camera; (2) visible‐near infrared objective lenses for the Pika L camera with a focal length of 17 mm, field of view of 17.6° and instantaneous field of view of 0.71 mrad; (iii) a global positioning system (GPS)/inertial measurement unit (IMU) flight control system for multi‐rotor aircraft, to record sensor position and orientation; and (iv) the Resonon hyperspectral data analysis software (Spectronon Pro, Resonon Inc.) with an ability to correct the GPS/IMU data using a georectification plugin. During the 2021–2023 breeding cycles, the hyperspectral‐equipped UAV was flown twice: the first flight occurred approximately 5–7 days after most lines had reached the heading stage, and the second was carried out about 2 weeks later. These specific time points were selected because of the critical importance of the late growth stages for determining GY. Research has shown that traits measured during certain key growth stages are often most predictive of GY across environments (Babar et al., 2006), as exemplified in Guo, Pradhan et al. (2020) where NDVI and other traits were shown to be most predictive of GY when taken at specific times during the plant growth cycle. To reduce noise and capture temporal variation, the HSI data from both flights were averaged. This involved first aligning the data from the two flights to ensure that the same plots were being compared, followed by correcting for any differences in lighting or atmospheric conditions. Finally, the spectral reflectance values for each wavelength were averaged across both flights to generate a more stable and representative dataset for each plot. UAV flights were conducted at a standard altitude of 60 m with a flight speed of ∼1.5 m/s for all years in the study. The hyperspectral sensing system covers a 380–1020 nm range divided into 300 bands. For the HSI collection, a front overlap of 85% and a side overlap of 70% were used. A white calibration tarp is also placed in the region of data collection to be used to calibrate the spectral data collected later in the processing pipeline. As mentioned, the high‐dimensionality spectral data are calibrated and analyzed using the Spectronon software (Spectronon Pro, Resonon) along with georectification and radiometric corrections to get the GPS/IMU and radiometric data. The regions of interest are manually selected based on each plot position, and the spectral data can be collected and exported for further analysis. The workflow of hyperspectral data collection is outlined and visualized in Figure 2.

### 2.4 Genotypic data collection

High‐quality DNA was isolated from freeze‐dried, powdered leaf tissue (∼100 mg) collected from 2‐week‐old plants using a modified cetyltrimethylammonium bromide (CTAB) protocol (Doyle & Doyle, 1987). Genotyping‐by‐sequencing (GBS) libraries were then constructed using specialized restriction enzymes, as described by Poland et al. (2012). After digestion, fragmented DNA samples were ligated with barcoded sequencing adapters and sample libraries with unique barcodes were sequenced on Novaseq 6000 SP flowcells. The sequencing process had a read length of 100 base pairs (Illumina Inc.) and the returned sequencing reads were properly aligned to the International Wheat Genome Consortium (IWGSC, 2014) RefSeqv1.0 reference genome with the use of the Burrows‐Wheeler Aligner version 0.7.12 (Li et al., 2009). This reference genome may be found at https://wheat‐urgi.versailles.inra.fr/Seq‐Repository/Assemblies. SNP identification and calling were implemented using the TASSEL 5GBSv2 pipeline version 5.2.35, developed by Glaubitz et al. (2014). All involved genotypes in the study were genetically characterized through GBS, identifying SNPs across the whole genome. Markers with more than 80% missing data, minor allele frequencies below 0.05, and heterozygosity larger than 10% were filtered away and discarded to guarantee a high‐quality genomic dataset. Imputation was performed using Beagle version 5.2 (Browning & Browning, 2016) program to handle missing data by using *k*‐nearest neighbor imputation to fill in missing genotypic data. The genetic dataset for each year consisted of roughly 23,000 SNP markers, offering a thorough genetic profile for the various wheat genotypes being examined in the study.

### 2.5 Analysis of phenotypic data

Analysis of variance (ANOVA) tests were conducted for each year in which data were gathered from the germplasm panel, assuming a mixed linear model. The R software environment (R Core Team, 2022) was used for this analysis, specifically employing the “lme4” package to fit the mixed‐effects models and perform ANOVA (Bates et al., 2016). Additionally, the “emmeans” package (Lenth, 2021) was used to calculate the best linear unbiased estimates (BLUEs) for GY and each of the hyperspectral wavelength bands. This was done using the given equation, with blocking considered as a random effect and genotype as fixed

$$Y_{ijkl}=\mu +g_{i}+E_{j}+gE_{ij}+R_{k}+B_{l\left(k,j\right)}+e_{ijkl}$$

This model includes an environment (year) component to account for variability due to changes in growing conditions across different years. Here we have $Y_{ijkl}\;$ as the observed value for the trait of interest for the *i*th genotype in the *l*th block, within the *k*th replicate, and *j*th environment; μ is our overall mean of the observed trait across all genotypes and blocks; the genotypic effect $g_{i}$ is the contribution of the *i*th genotype to the observed trait, deviating from the overall mean μ; $E_{j}$ is the effect of environment *j* (where environment refers to the different years in which the trials were conducted); $gE_{ij}$ is the *j*th genotype by *i*th environment interaction effect; $R_{k}$ is the replicate effect for the *k*th replicate, representing variability between replicates, which could arise due to large‐scale field heterogeneity; $B_{l(k,j)}$ is the block effect within the *k*th replicate and *j*th environment for the *l*th block; and $e_{ijkl}$ represents the residual variation that is not explained by the model's other terms. The final two terms are assumed to follow an independent and identically distributed normal distribution with a mean of zero and a constant variance such that $B_{l(k,j)}∼N\left(0,\sigma_{B}^{2}\right)$ and $e_{ijkl}∼N\left(0,\sigma_{e}^{2}\right)$. The BLUEs were calculated with DTH as a covariate to control for the confounding effect that differences in maturity can have on influencing prediction outcomes for the models. This method follows the approach outlined in Guo, Pradhan et al. (2020), ensuring that maturity does not obscure the relationships between these traits. The variances for each effect were obtained to compute *H* ^2^ for yield within each year, and for each hyperspectral wavelength region, all the model terms were considered as random, except by the common mean μ with $g_{i}∼N\left(0,\sigma_{G}^{2}\right)$ and it was determined by:

$$H^{2}=\frac{\left(\sigma_{G}^{2}\right)}{\left(\sigma_{G}^{2}+\frac{\sigma_{e}^{2}}{r}\right)}\times 100$$

The heritability estimates were calculated separately for each year (environment), with two replicates per genotype included in the analysis, as changes in growing conditions between years represent different environments that impact phenotypic expression. In this equation, $\sigma_{G}^{2}\;$ represents the genotypic variance, and $\sigma_{e}^{2}$ is the residual variance is crucial for comprehending the fraction of overall phenotypic variation that may be attributed to genetic disparities among the genotypes. By deriving the *H* ^2^, it is possible to independently assess the data outcomes of each year. This helps to better quantify genetic variation and understand the impact of changes in the growing environment from one year to the next. Later, this allows for a better picture of the interaction between environment and genotype across time.

### 2.6 Prediction modeling approaches

#### 2.6.1 Genotypic effects model (G)

Throughout the study, “environment” refers to the growing conditions experienced during a specific year. Each year represents a distinct environment due to changes in weather, temperature, and other external factors. This definition applies consistently throughout all models described below. The first model (M1) relies on genomic data to predict GY and serves as the baseline to compare the different models employed for predicting within a year for a single environment. This model corresponds to the reproducing kernel Hilbert space (RKHS) framework, implemented using the BGLR package (Pérez & de Los Campos, 2014), which has been extensively applied in GP for various crops and traits, demonstrating its robustness and predictive power (Crossa et al., 2017; Heslot et al., 2012; VanRaden, 2008). It attempts to explain yield performance ($Y_{i}$) of the *i*th genotype incorporating a common effect μ across different wheat lines plus genomic ($g_{i}$) effects and an error term ($\varepsilon_{i}$) as follows:

(M1) $$Y_{i}=\mu +g_{i}+\varepsilon_{i}$$

Here, $Y_{i}$ is the BLUE for GY for the $i^{\mathrm{th}}$ genotype; g is the vector of genomic effects $g=\{g_{i}\}$ following a multivariate normal distribution so that g∼ N(**0**, **G** $\sigma_{g}^{2}$) where $G=\frac{XX^{\prime }}{p}$ denotes the genomic relationship matrix and **X** being the standardized and centered (by columns) matrix of *p* SNPs, $\sigma_{g}^{2}$ is the corresponding variance component; and $\varepsilon_{i}∼N\left(0,\sigma_{\varepsilon }^{2}\right)$ with $\sigma_{\varepsilon }^{2}$ as the variance component of the error term. The correlation between the effects of the random variables is given by the off‐diagonal elements of matrix **G**, providing the opportunity to transfer information between several lines which in turn could allow the capability to predict the performance of lines that have not been assessed in any field trial.

#### 2.6.2 HSI effects model (H)

The HSI effects, phenomics‐based model (M2), is not unlike the previous but instead of having SNP marker covariates to build the genomic relationship matrix **G**, it relies on incorporating phenotypic hyperspectral waveband information from the HTP data kernel (H)

(M2) $$Y_{i}=\mu +H_{i}+\varepsilon_{i}$$

where $H_{i}$ represents the hyperspectral waveband main effect of the *i*th genotype. Where the joint distribution of the involved traits is modeled such that $H=\left\{H_{i}\right\}∼N\left(0,P\sigma_{P}^{2}\right)$, where $P=\frac{SS^{\prime }}{m}$ is the hyperspectral‐derived relationship matrix with **S** as a matrix made up of the centered and standardized BLUE values of the *m* hyperspectral wavebands and $\sigma_{P}^{2}$ denotes the corresponding variance component. Models incorporating HSI data have been demonstrated to improve GP by capturing phenotypic variation not accounted for by genomic data alone, particularly for complex traits like GY (Krause et al., 2019). Like the previous model, it is meant for within year predictions and it does not account for environmental effects.

#### 2.6.3 Genotypic + hyperspectral model (G + H)

This model combines the two previous models to contain both the genomic and the HSI data kernels. It allows the appraisal of how combining HTP data with traditional genomic approaches can influence prediction accuracies within a single year:

(M3) $$Y_{i}=\mu +g_{i}+H_{i}+\varepsilon_{i}$$

All of the model terms have been defined in the previous two models.

#### 2.6.4 Environment (year) interaction models

This model (G + G **×** E) extends the genomic effect only model by incorporating the environmental and interaction G×E effects

(M4) $$Y_{ij}=\mu +E_{j}+g_{i}+gE_{ij}+\varepsilon_{ij}$$

where $Y_{ij}$ is the BLUE for GY for the $i^{\mathrm{th}}$ genotype in the $j^{\mathrm{th}}$ environment (year); the $E_{j}$ component represents the effect of the environment (year) in which the trial was conducted.; $g_{i}$ is the genomic main effect as previously described; $gE_{ij}$ corresponds to the interaction between the $i^{th}$ genotype with $j^{th}$ environment (year). It is modeled by accounting for the interaction between each molecular marker and each environment. From results from the multivariate normal distribution the vector of interactions is modeled as in Jarquin et al. (2014) such that $gE=\left\{gE_{ij}\right\}∼\mathrm{MN}\left(0,Z_{g}GZ_{g}^{T}⊗Z_{E}Z_{E}^{T}\;\sigma_{gE}^{2}\right)$where $Z_{g}$ and $Z_{E}$ represent incidence matrices that connect the phenotypes with the genotypes and environments, respectively, and $\sigma_{gE}^{2}$ is the corresponding variance component. Finally, $\varepsilon_{ij}$ is the residual error addressing non‐explained variability by any of the previous model terms, such that $\varepsilon_{ij}∼N\left(0,\sigma_{\varepsilon }^{2}\right)$ with $\sigma_{\varepsilon }^{2}$ as the variance component of the error term. With the inclusion of these environmental effects, the differences between years and across seasons can be better captured.

This model (M5) extends the hyperspectral effect model (M2) by incorporating environmental and hyperspectral‐by‐environment interaction (H×E) effects:

(M5) $$Y_{ij}=\mu +E_{j}+H_{ij}+HE_{ij}+\varepsilon_{ij}$$

where $H_{ij}$ represents the hyperspectral effect of the $i^{\mathrm{th}}$ genotype in the $j^{\mathrm{th}}$ environment, modeled to capture the variability in hyperspectral data. The $HE_{ij}$ term represents the HTP vector of interactions between the $i^{th}$ genotype by $j^{th}$ environment (year) modeled with aerially derived hyperspectral data. Guo, Pradhan et al. (2020) demonstrated the utility of similar phenotypic trait‐derived relationship matrices for capturing complex environmental interactions, showcasing their potential to improve predictions of GY in wheat. It is assumed a multivariate normal distribution so that $HE=\left\{HE_{ij}\right\}∼N\left(0,Z_{g}HZ_{g}^{T}⊗Z_{E}Z_{E}^{T}\sigma_{HE}^{2}\right)$ where $\sigma_{HE}^{2}$ represents the corresponding variance component (Jarquin et al., 2014). In addition, all terms previously presented were combined into a single model that contains genotypic, HTP, G × E, and H × E effects (G + H + G × E + H × E) as follows:

(M6) $$Y_{ij}=\mu +E_{j}+g_{i}+H_{ij}+HE_{ij}+gE_{ij}+\varepsilon_{ij}$$

#### 2.6.5 Environment interaction models with ECs

Three more models were tested, including a kernel for the ECs, in addition to those described above. The relationship matrix for this kernel was constructed similarly to the hyperspectral relationship matrix with the idea that incorporating weather covariates can help the models better account for variations from 1 year to the next. The first of these three models (G + w + G × E + G × w) contains the genetic main effect (G), the environment effect (E), the ECs main effect (w), and the interaction effects G × E and G × w:

(M7) $$Y_{ij}=\mu +E_{j}+g_{i}+w_{j}+gw_{ij}+gE_{ij}+\varepsilon_{ij}$$

where the term $w_{j}$ is the ECs kernel effect for the *j*th environment assuming a joint distribution such that $w=\left\{w\right\}∼N\left(0,K\sigma_{K}^{2}\right)$, where $K=\frac{WW^{\prime }}{q}$ is the ECs‐derived relationship matrix with **W** as a matrix made up of the values of the *q* ECs and $\sigma_{K}^{2}$ signifies the corresponding variance component. $gw_{ij}$ corresponds to the interaction effect between each marker SNP and each environmental covariate. This model term is modeled assuming a multivariate normal distribution such that $gw=\left\{gw_{ij}\right\}∼N\left(0,Z_{g}HZ_{g}^{T}⊗Z_{E}KZ_{E}^{T}\sigma_{gw}^{2}\right)$.

The second model of this series replaces the genotypic effects with the HSI effects (H + w + H × E + H × w):

(M8) $$Y_{ij}=\mu +E_{j}+H_{ij}+w_{j}+HE_{ij}+Hw_{ij}+\varepsilon_{ij}$$

Finally, a comprehensive model of all effects available was tested

(M9) $$\begin{array}{ccc} Y_{ij} & = & \mu +E_{j}+g_{i}+w_{j}+H_{ij}+HE_{ij}+Hw_{ij}+gE_{ij}\\ & & +\;gw_{ij}+\varepsilon_{ij} \end{array}$$

With the comprehensive model, we hope to improve the precision of predicting wheat line performance by integrating genetic, hyperspectral, and environmental data, as well as their interactions. All models (M1–M9) were implemented using the CHiDO software, an advanced GP platform designed for efficient integration of omics data in predictive frameworks (González et al., 2024). CHiDO leverages the BGLR package (Pérez & de Los Campos, 2014) as its underlying framework for model execution. This approach facilitates the seamless application of Bayesian linear regression models to GP tasks, where for the purposes of this research, an RKHS model was used.

#### 2.6.6 Machine learning modeling approaches

Several ML algorithms (random forest regression [RFR], support vector machine regression [SVMR], gradient boosting regression [GBR], and artificial neural network [ANN]) were also tested to predict GY by integrating genetic (G), hyperspectral (H), and environmental data (w). Each model has distinct strengths in capturing the complex interconnections within the data. All model implementations were carried out using Python, leveraging libraries such as scikit‐learn for model development and evaluation (Pedregosa et al., 2011), NumPy for numerical computations (Harris et al., 2020), and pandas for data manipulation (McKinney, 2010). The GridSearchCV module from scikit‐learn was used extensively to optimize hyperparameters for all models. The selection of SVMR was made because of its effectiveness in dealing with datasets that have many dimensions and a limited number of samples. SVMs are flexible models that can handle both linear and nonlinear classification by mapping data into higher dimensional spaces. This allows for classification and regression even with noisy data (Basak et al., 2007). Here, the radial basis function (RBF) kernel was used to handle nonlinear correlations. The hyperparameter grid for RBF optimization included *C* values of [0.1, 1, 10, 100] and γ values of [0.001, 0.01, 0.1, 1], ensuring a comprehensive search across penalty and kernel width values. The purpose of this kernel is to input characteristics into higher dimensional spaces, thus enabling linear separation. A grid search was conducted to optimize hyperparameters such as the penalty parameter (*C*), which balances model simplicity with error minimization, and the kernel width (gamma), which determines the influence of individual training examples.

RFR was also employed, which is a machine learning technique that creates multiple decision trees, with each tree trained on a randomly selected subset of the data. RFR aggregates numerous decision trees to enhance prediction accuracy and mitigate overfitting risks (Probst et al., 2019). For RFR, the hyperparameter grid included the number of trees (n_estimators) ranging from [100, 500, 1000], and the number of features considered at each split (max_features) ranging from [auto, sqrt, log2]. Maintaining model diversity through choosing a random sample of predictors at each split. A grid search was carried out to determine the optimal number of trees (n_estimators) and the number of features considered at each split (max_features), effectively balancing variance and bias to ensure stable and consistent performance.

GBR employs an ensemble of weak learners, typically decision trees, to create a strong predictive model. GBR iteratively improves prediction accuracy by targeting errors made by earlier models (Chen & Guestrin, 2016). It builds a sequence of trees, with each tree correcting the errors of its predecessors, enabling the model to capture intricate and nonlinear interactions. The grid search for GBR included the following hyperparameter ranges: learning rates [0.01, 0.1, 0.2], maximum tree depth values [3, 5, 7], and the number of estimators [50, 100, 200]. Several parameters were fine‐tuned for this approach, such as the number of estimators, learning rate, maximum tree depth, and minimum samples necessary for node splitting and leaf node formation.

ANNs were the final ML method tested to simulate complex relationships between predictors and GY. ANNs consist of layers of connected neurons, where the weight of each connection is adjusted during training to reduce error (Schmidhuber, 2015). The ANN architecture was optimized by exploring various configurations, including one to three hidden layers, with each layer containing 32, 64, or 128 neurons. The activation functions tested included rectified linear unit (ReLU) and sigmoid for hidden layers and linear for the output layer. The optimizer was chosen from Adam, SGD, and RMSprop, and the learning rate was adjusted over a range of [0.001, 0.01, 0.1]. Early stopping was implemented to prevent overfitting. The hidden layers of the ANN models used the ReLU activation function, thereby introducing non‐linearity and enabling the network to acquire intricate patterns within the dataset, with the output layer using the linear activation function to predict continuous values. The ANN was trained using the backpropagation technique, adjusting the weights by gradient descent to minimize the mean squared error. The architecture and hyperparameters, such as the number of hidden layers and neurons, were optimized to attain optimal performance.

### 2.7 Cross validation and forward prediction

Cross validation for each model was carried out to ensure accurate and impartial performance evaluations. K‐fold CV using 10 folds was carried out where the dataset was partitioned into 10 equal subsets, giving a thorough assessment of the stability and generalization capacity of each model (Guo, Pradhan, et al., 2020). The performance of each model was measured with the Pearson correlation coefficient (*ρ*). A discriminant analysis of principal components (DAPC) was performed separately for each year to account for genetic relatedness across lines (Jombart et al., 2010). From the DAPC results, the population was categorized into 10 clusters, of which a random selection of lines was used to create five CV subgroups. This is done so that no lines within the same cluster are included in both the training and validation sets, allowing for the appropriate management of relatedness during model training. The CV was conducted 10 times, using 80% of the phenotypic data for training and the remaining 20% for testing. The machine learning modeling was conducted using the Python programming language, making use of libraries like scikit‐learn and TensorFlow to streamline the implementation process.

There were two distinct CV approaches to evaluate the predictive performance of the models. Data from the three consecutive years collected at the Citra location were used to assess the models' accuracy in predicting GY both within a single year and across multiple years. The within‐year predictions were evaluated using a CV2 approach (Jarquin et al., 2017), utilizing the 10‐fold CV as mentioned. The dataset for each year was divided into 10 equal subsets (folds). Each fold was used as a validation set once, while the remaining nine folds formed the training set. This process was repeated 10 times, ensuring that each fold served as the validation set once. The model was trained on the K‐1 folds and used to predict the GY for the validation fold, providing a robust evaluation of the model's stability and generalization ability within the same year. To assess the models' ability to predict GY across years, a forward prediction approach was applied, leveraging data from earlier years to predict subsequent years. Specifically, data from 2021 were used to predict GY for 2022, while data from 2021 and 2022 were combined to predict GY for 2023. This approach is similar to the CV0‐Y framework described by McBreen et al. (2024), which utilizes iterative CV to predict performance in untested years. The approach in this research evaluates prediction accuracy by designating specific years as the validation set, simulating a practical breeding scenario where previous and current data are utilized to predict future performance. Unlike CV schemes with random partitioning and multiple iterations, this approach uses a single validation set for each forward prediction scenario (e.g., 2021 to predict 2022, or 2021 and 2022 to predict 2023). Consequently, no error bars are included, as predictions are based on fixed training and validation sets rather than repeated random splits.

The Pearson correlation between anticipated and observed values within each environment was computed to get a dependable measure of model performance on an annual basis. Utilizing the methods introduced by Tiezzi et al. (2017) and implemented by Canella Vieira et al. (2022), a weighted average correlation coefficient across environments was computed. The weighted averaged correlation considers the variability in sampling and the number of observations for each of the specific settings and was computed with the following formula:

$$r_{\varphi }=\frac{\sum_{l=1}^{11}\frac{r_{j}}{V\left(r_{j}\right)}}{\sum_{l=1}^{11}\frac{1}{V\left(r_{j}\right)}}$$

where $V\left(r_{j}\right)=\frac{1\;-\;r_{j}^{2}}{n_{j\;}-\;2}$ and $n_{j}$ is the number of genotypes at the *j*th environment. This methodology accounts for the variability and uncertainty inherent in each environment.

The coincide index (CI) was created to evaluate the ability of different models to reliably identify the top‐performing genotypes within a given forward prediction scenario. Specifically, the CI measures the proportion of genotypes that are common between the predicted and observed top 25% of grain‐yielding lines, providing a practical measure of a model's usefulness in a breeding context where the objective is to prioritize the selection of superior genotypes. To calculate the CI, the predicted rankings of genotypes were compared against their observed rankings for GY. In this analysis, the CI was calculated for the forward prediction scenario where data from 2021 and 2022 were used to predict GY in 2023. The metric provides insight into how well models identify superior‐performing genotypes, which is a critical goal in breeding programs.

## 3 RESULTS

### 3.1 Descriptive statistics and heritability

Table 2 displays descriptive statistics and *H* ^2^ for GY across the three growing years (2021–2023) at the PSREU in Citra, FL. Fluctuations in GY can be seen from 1 year to the next, with the highest mean in 2021 for an average GY of 2773 kg ha^−1^. The following year presented decreases in GY at an average of 2582 kg ha^−1^, with an even further decrease in the final year, 2023, resulting in at a mean of 2149 kg ha^−1^. These variations in yield across years highlight the impacts of environmental conditions, as well as different germplasm sets and population sizes. Despite these differences, the observed GY was consistently lower than the global average of 3300 kg ha^−1^. This can be attributed to the fact that Citra is considered a heat‐stressed environment for wheat. Hence, the lines with consistently high GY in this environment can be considered as heat stress tolerant. The standard error (SE) for GY remained regularly stable over the 3 years, suggesting a persistent degree of precision in yield estimation. From 2021 to 2023, the broad‐sense heritability (*H* ^2^) for GY ranged from 54.3% to 62.3%, reflecting some variability across years. The lowest *H* ^2^ value was observed in 2021, while the highest was recorded in 2022, highlighting some variation in heritability estimates across years, perhaps reflecting differences in environmental conditions and population dynamics. Importantly, this variability suggests that not all variance can be attributed to random error, as the observed heritability reflects a measurable genetic influence on GY under varying conditions. There was some variation from year to year for GY, but it remained somewhat high, never dipping below 50%, demonstrating a relatively high genetic influence on GY overall.

**TABLE 2:** Descriptive statistics and heritability (*H* ^2^) of grain yield (GY) in kg ha^−1^ for 2021–2023. Summary of the average sample size, GY in kg ha^−1^ mean and standard error (SE), range, and average heritability (*H* ^2^) for each site across multiple years (2021–2023).

Site	Year	Sample size	GY (mean ± SE)	GY range	*H* ^2^ (%)
Citra, FL	2021	330	2773 ± 55	702–5441	54.3
Citra, FL	2022	262	2582 ± 47	1643–4532	62.3
Citra, FL	2023	139	2149 ± 76	690–4725	61.3

*Note*: Aggregated data for Citra, FL, are presented in the table, which illustrate the performance and variability of grain yield at this site across years

**FIGURE 3:**
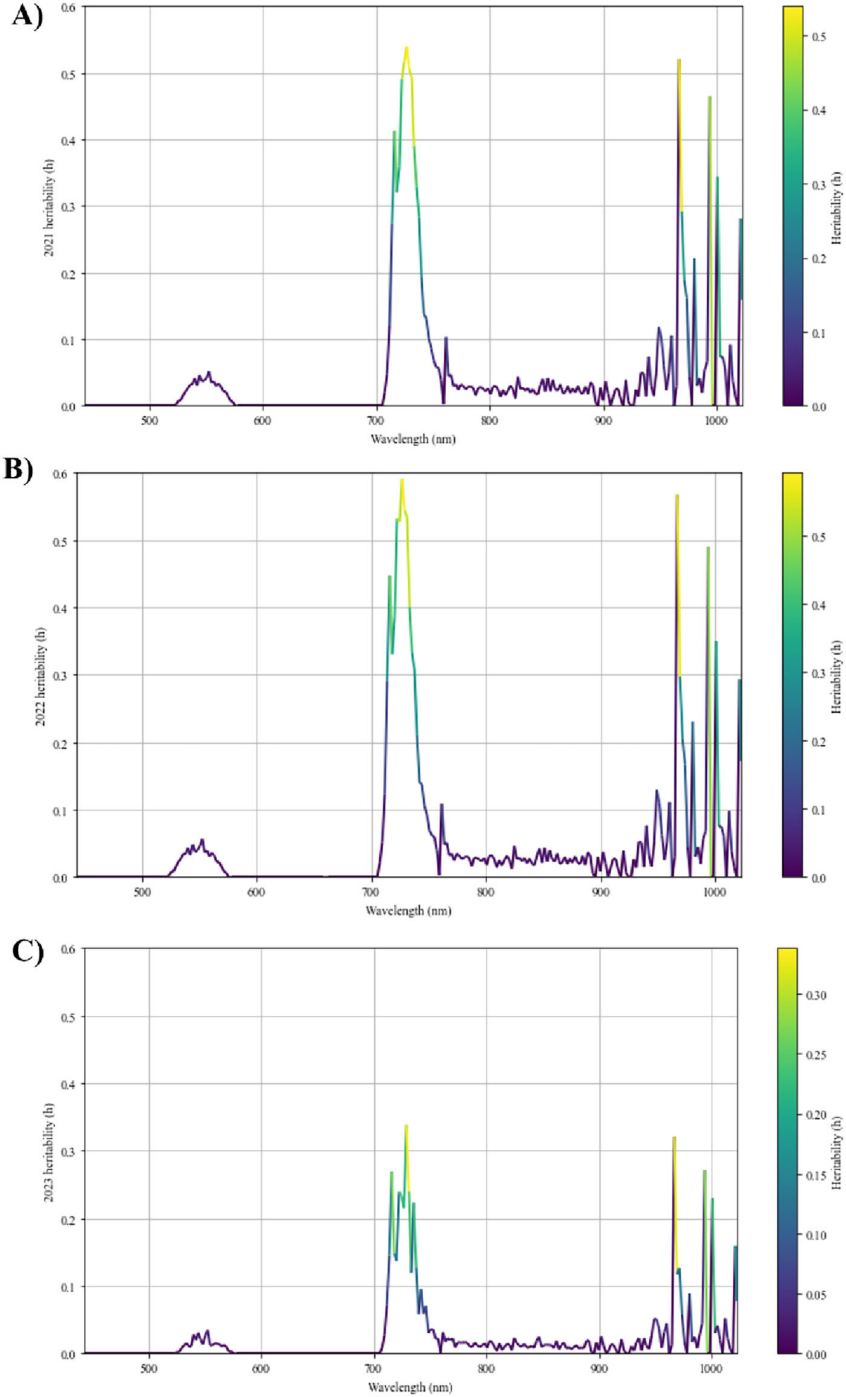
Broad sense heritability values for hyperspectral wavebands for 2021–2023. The broad sense heritability (*H* ^2^) values for hyperspectral wavebands measured during the three growing seasons of (A) 2021, (B) 2022, and (C) 2023 at the Citra, FL, site. Each plot displays the *H* ^2^ values for different hyperspectral wavelengths, with colors indicating different levels of heredity, with data derived from the mean hyperspectral wavelength values for each corresponding year.

Two spectral data acquisition flights were conducted per year both at or near solar noon. Figure 3 illustrates the heritability values for the hyperspectral wavebands for each of the 3 years. These values represent the averaged heritability estimates derived from two separate flight datasets collected during each year. Each plot within the figure shows the *H* ^2^ value across the entirety of the wavelength region collected (∼380–1020 nm), with color gradients used to indicate different levels of heritability. These wavelength values for each specific year were used to give a view of the genetic effect over the whole spectrum of wavelengths. The peaks seen in each plot show heritability hotspots where wavebands in these regions are more likely to have higher heritability. Trends can be seen over the years, with certain wavelengths consistently exhibiting higher heritability, which may reflect biological interactions such as differential absorption or reflection rather than solely genetic stability. For 2021, there is a pronounced peak at around the 700 nm wavelength mark, with similar heritability peaks observed in the 2022 and 2023 data, albeit with variation in the exact wavelengths and magnitudes of heritability. Regardless of differences in their magnitudes, the similarity in heritability peaks for the wavelength regions suggests that data were also uniformly collected and processed between years.

To better understand the environmental impact on GY and capture the genotype–environment interaction (G × E) across years, we also included ECs in the modeling. Citra experienced moderate to high rainfall, consistently high humidity levels, and recurrent temperatures exceeding 30°C during the grain‐filling stages. The temperature weather data for each growing season are depicted in Figure 2. There were also fluctuations in solar radiation and minimum and maximum daily temperatures as well as variability in rainfall across the years (Table 1).

### 3.2 GY prediction

**FIGURE 4:**
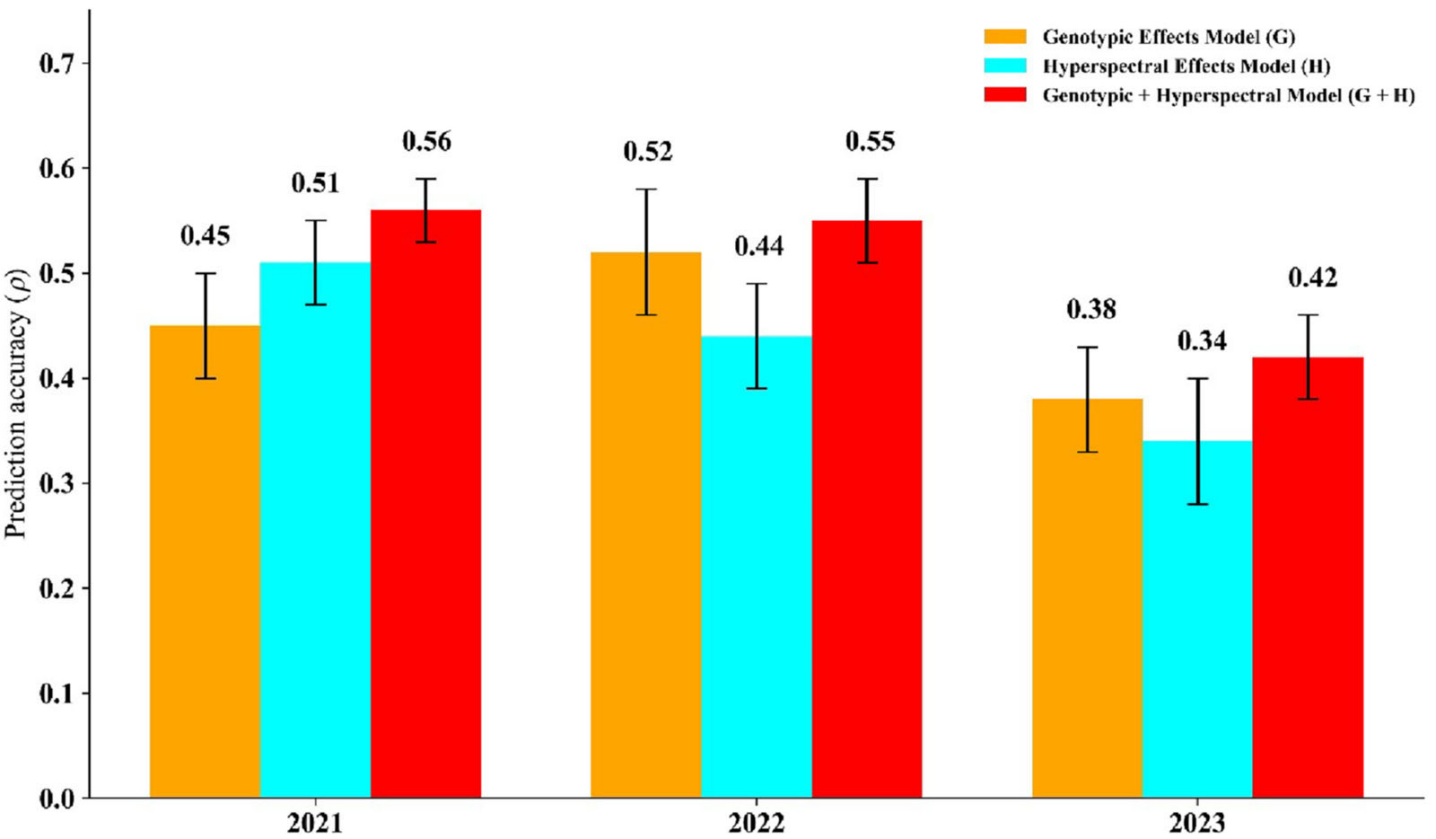
Within‐year prediction accuracy of G, H, and G + H models in predicting grain yield (GY). Model prediction accuracies for GY (kg ha^−1^) by year and model (Citra, FL). The bar graph displays the correlation (*ρ*) between actual and predicted GY for different models applied to lines from Citra, FL, over 2021–2023. Each model's performance is validated using a 10‐fold cross‐validation approach with an 80/20 training‐testing split. The models are G (genomic data only), H (hyperspectral data only), and G + H (genomic and hyperspectral data).

The model's predictive ability to predict GY genotype performance was first evaluated within each season/growing year. Several models were evaluated including the genomic effects model (M1), HSI effects model (M2), and genotypic + hyperspectral model (M3) under the CV2 cross‐validation scheme predicting incomplete field trials (Figure 4), along with different ML approaches that integrated combinations of these omics. The error bars in Figure 4 represent the standard error (SE) of prediction accuracy (*ρ*) derived from CV folds. The SE of the mean difference in performance metrics across folds was computed by considering the dependencies introduced by overlapping training and test sets in *k*‐fold CV. This calculation incorporates the ratio of test to training samples to account for the non‐independence of folds, ensuring an accurate estimation of variability in model performance (Bouckaert & Frank, 2004). Model accuracy was measured by the Pearson correlation coefficient (*ρ*) between the actual and the predicted values. For the 2021 year, the G model (M1) was outcompeted by its hyperspectral counterpart (M2), achieving *ρ* values of 0.45 and 0.51, respectively. When the two data types were combined into a single model (M3), a further increase was observed to 0.56. In the following year, 2022, the performance of the two models was inverted with the G model (M1) coming in at 0.52 and H (M2) at 0.44. Again, the G + H model (M3) outperformed both models (*ρ *= 0.55). For the third and final year, 2023, the predictive capabilities dipped all around but the trends in predictive abilities maintained from the previous year. The ML models that were tested contained both HSI and SNP marker data as well as just HSI data in order to see if hyperspectral data alone could achieve accurate predictions within a season.

**FIGURE 5:**
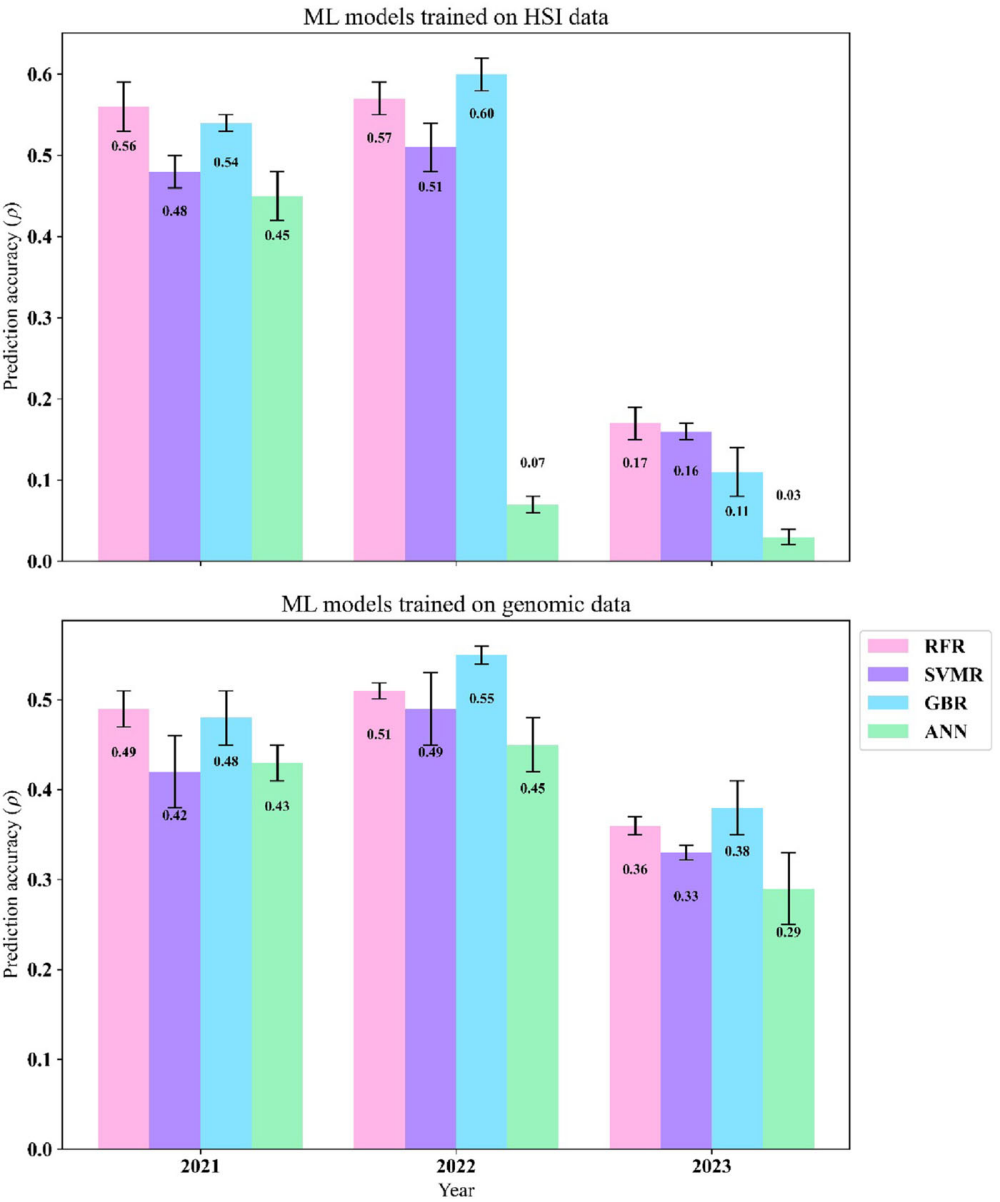
Bar plots of grain yield (GY) prediction accuracy using machine learning (ML) models trained on hyperspectral imaging (HSI) or genomic data. Bar graph illustrating prediction accuracies for GY values (kg ha^−1^) for Citra, FL, using the random forest regressor (RFR), support vector machine regressor (SVMR), gradient booster regressor (GBR), and artificial neural net (ANN) models when they are trained on HSI or genomic data to predict GY.

The ML‐based models that made predictions using the HSI data alone performed competitively in the first year to the highest performing model that combined both sources of information with RFR, matching the predictive performance of the M3 (*ρ *= 0.56). It was closely followed by GBR at 0.54 and trailed by SVMR and ANN (0.48 and 0.45) as seen in Figure 5.

These models performed similarly for the following year, with the GBR accuracy (*ρ *= 0.60) even outcompeting the M3 model (*ρ *= 0.55). However, the ANN model was an exception, as its performance declined after the first year. In 2023, the model, incorporating SNP marker data, outcompeted the ML models when relying on HSI only, with the G, H, and G + H models (M1–M3) achieving accuracies of 0.38, 0.34, and 0.42, respectively. Meanwhile, the ML models struggled this year, with none being able to reach an accuracy above 0.20 (Figure S1). When these same models are trained on the genomic data rather than the HSI data, they perform similarly but slightly better than the G‐only model (M1) with the highest accuracies from RFR in 2021 at 0.49, and from GBR in 2022 and 2023 at 0.55 and 0.38, respectively.

**FIGURE 6:**
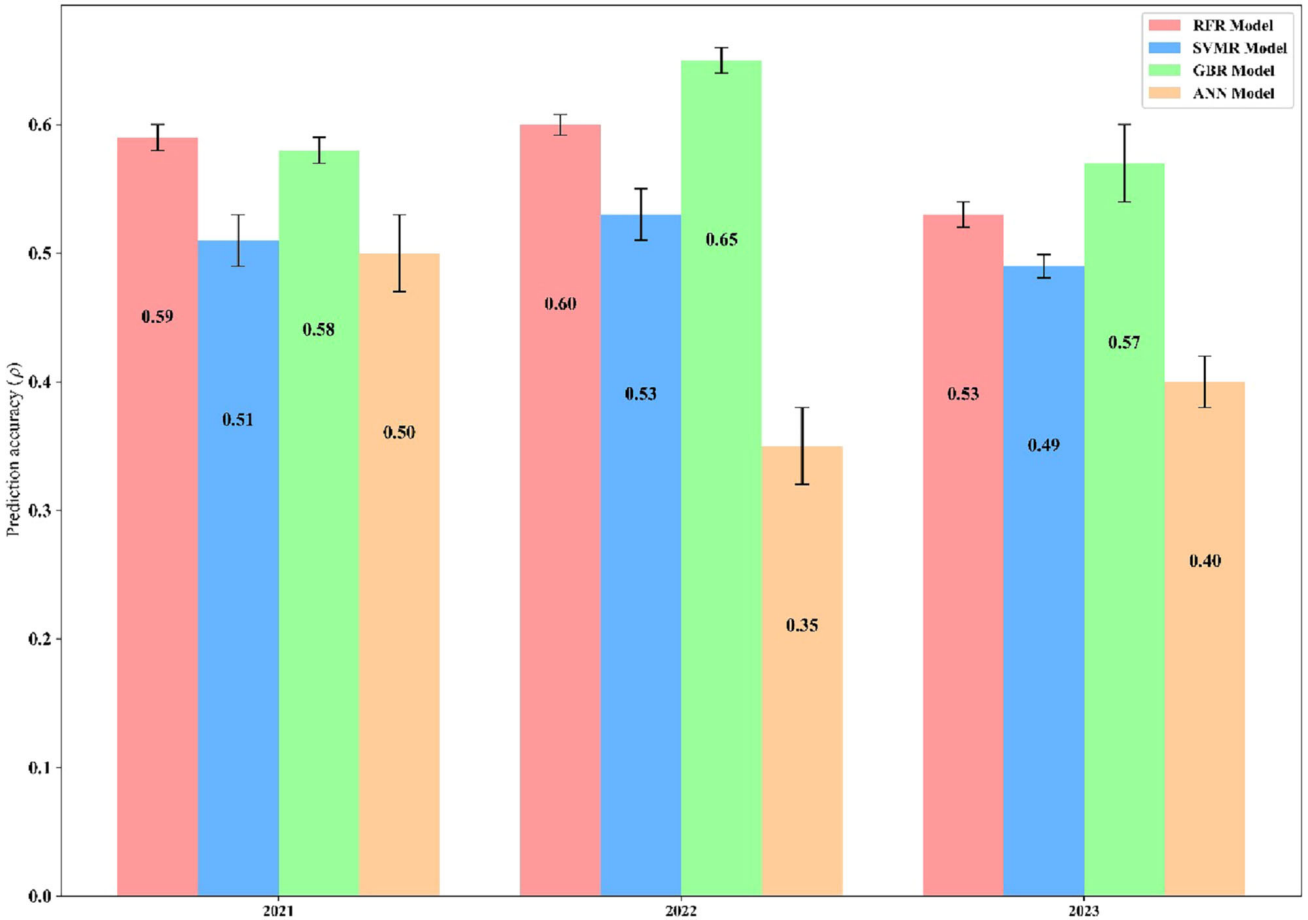
Within‐year prediction accuracy of machine learning (ML) models incorporating hyperspectral and genomic data in predicting grain yield (GY). Model prediction accuracies for GY by year and model. Displays the correlation (*ρ*) between actual and predicted GY for ML models applied to lines from Citra, FL, from 2021 to 2023. Model performance is validated using a 10‐fold cross‐validation approach with an 80/20 training‐testing split. Models include random forest regression (RFR), support vector machine regression (SVMR), gradient boosting regression (GBR), and artificial neural network (ANN). Error bars represent the standard error (SE) of the prediction accuracies.

When SNP marker data were added to the ML models along with the HSI data for the within‐year predictions, prediction accuracies increased to levels that matched or, in some cases, surpassed the M3 approach, as illustrated in Figure 6. In 2021, the GBR and RFR models just outperformed the M3 model, and in 2022, they surpassed it, achieving accuracies of 0.65 and 0.60, respectively. In the third year, the GBR returned an accuracy of 0.57 and RFR of 0.53, maintaining similar predictabilities to previous years. While the M3 model produced a dip in accuracy in this year (*ρ *= 0.42), suggesting the ML model's ability to perform well in specific prediction years. These results highlight the consistent outperformance of the GBR and RFR models in predicting within years GY compared to the RKHS‐based M3 model. For within‐year predictions, M3 is a reliable model that integrates genomic and hyperspectral data, leveraging the genetic stability of SNP markers and the phenotypic detail of HSI. ML models like GBR and RFR excel in capturing nonlinear interactions when diverse, high‐quality datasets are available but can be more sensitive to environmental variability. Hyperspectral‐only models (M2) are less consistent and work best as complementary tools to genomic models (M1), highlighting the value of integrating diverse data sources for robust predictions.

### 3.3 Mixed effect models and across year forward prediction

**FIGURE 7:**
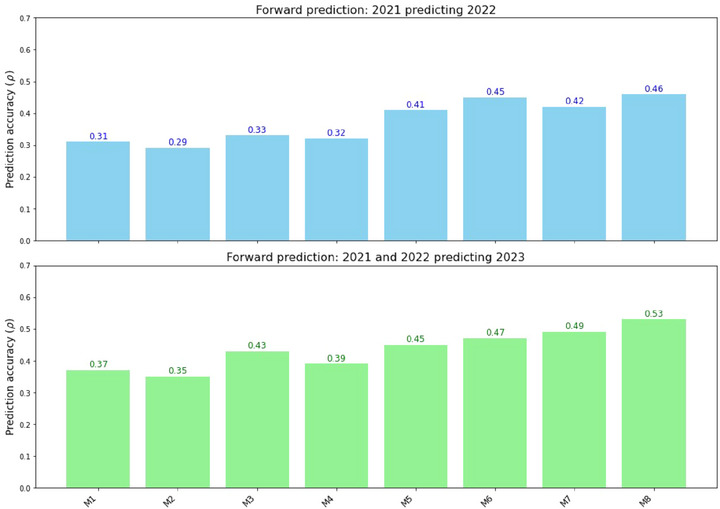
Forward prediction accuracies for grain yield (GY) using different reproducing kernel Hilbert space (RKHS)‐based models. Bar graphs illustrating the prediction accuracies (*ρ*) of different models for predicting GY in Citra, FL. The top plot shows the prediction accuracies for the 2022 growing season using data from a single year, Citra 2021. The bottom plot displays the prediction accuracies for the 2023 growing season using data from Citra 2021 and 2022.

A forward prediction scheme was implemented where data from earlier years were used for model calibration to predict GY for lines in subsequent years. For example, data from 2021 were used to predict GY for 2022, and data from both years were merged to predict 2023. While this strategy mimics a practical breeding scenario by using previous and present‐year data to anticipate upcoming years, it is important to note that HSI data were collected during the validation year itself, as this information is dependent on the lines being grown in the respective environment. This prediction scenario was carried out for both the semi‐parametric and ML models. Figure 7 shows that the trend seen both within and across years holds true for the forward prediction as well, with prediction accuracies increasing as more data sources and their interaction effects are added to the modeling. The M5 model showed the lowest accuracy (*ρ *= 0.29) and the comprehensive model containing all effects was the highest (*ρ *= 0.46) when 2021 was used to predict 2022. When data from both years were used to predict 2023, overall accuracies increased. However, the model ranking remained consistent, with the lowest accuracy at 0.35 and the highest at 0.53 with M9. This suggests that having more data from previous years can improve predictions for the following year, particularly when the location remains the same.

When the ML models were implemented integrating hyperspectral, genomic, and ECs data types, they performed generally worse overall compared to the RKHS‐based models across all evaluations (Figure S2). For forward predictions across years, comprehensive parametric models like M9 are the most reliable, as they integrate genomic, hyperspectral, and environmental covariate data while modeling genotype‐by‐environment interactions, making them well‐suited for capturing the complexities of multi‐year predictions under variable environmental conditions. However, other models, such as M1‐ or hyperspectral‐based approaches, can complement one another when certain data types are incomplete or unavailable, providing flexibility in leveraging available resources to improve predictive accuracy. Although utilizing a comprehensive model with many data types is ideal, combining the available data one has can still achieve improved predictions compared to the baseline model that relies solely on genomic information.

**FIGURE 8:**
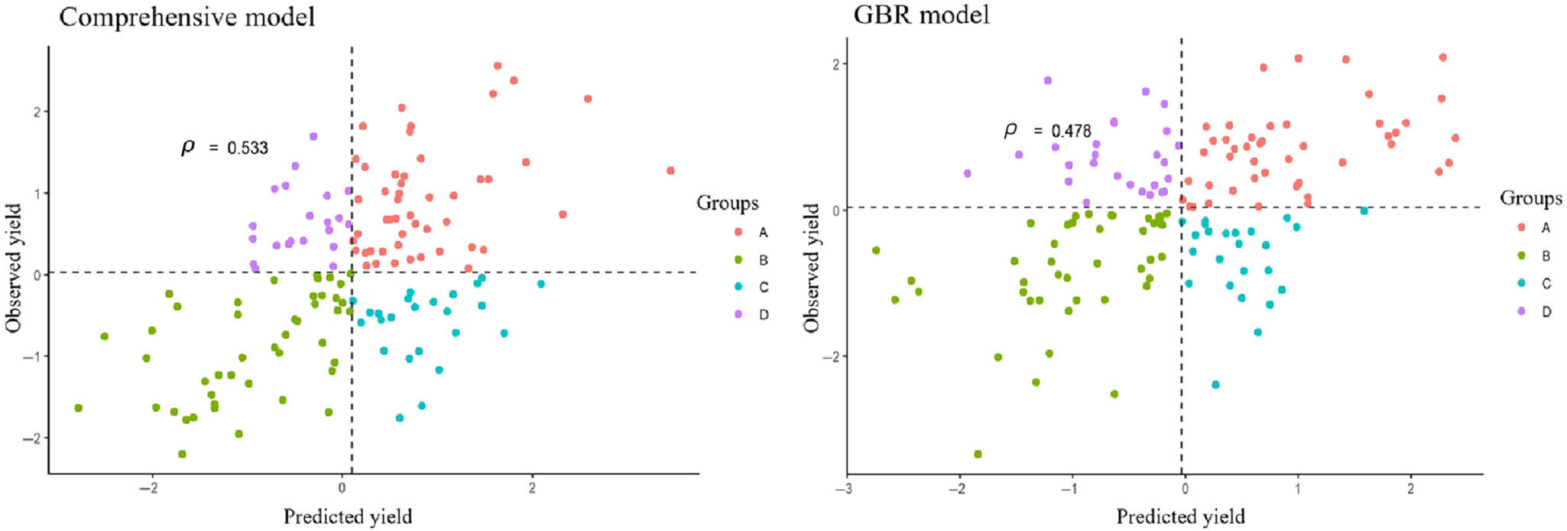
Model comparison for prediction of top 25% yielding lines in forward prediction. Scatter plot visualizing the predicted versus observed yield for the comprehensive multivariate and the gradient boosting regression (GBR) models under the forward prediction scenario where 2023 is predicted from 2021 and 2022 data. Dashed lines show mean observed and predicted grain yield (GY) values, indicating the central tendency of the data around 0. Quadrants A–D categorize data points based on their alignment relative to these mean values: predictions and observations above both means, below both means, above the mean prediction but below the mean observation, and below the mean prediction but above the mean observation.

A comparative analysis in which the reliability of the two best performing models predicting the top 25% yielding lines based on ranking using “2021 and 2022 to predict 2023” forward prediction scenario was done (Figure 8). In Figure 8, the dashed lines represent the mean values for both the predicted and observed yield, effectively splitting the data into four quadrants. These quadrants are used to categorize genotypes based on their performance relative to the average: those with predicted and observed yields both above or both below the mean, and those where predictions either overestimate or underestimate the actual performance. The CI was used to quantify the percentage overlap between the genotypes ranked in the top 25% for observed and predicted yields. The comprehensive model, which integrated multiple data sources, achieved a CI of 55% of the top 25% yielding lines, whereas the GBR model correctly identified 46% of these. The performance of the other ML models can be found in the Supporting Information (Figure S1). These results demonstrate the comprehensive model's superior predictive ability under the forward prediction context.

## 4 DISCUSSION

### 4.1 Comparison of genomic and hyperspectral prediction models

While both HSI and GP models offer valuable tools for breeding, their roles differ fundamentally in terms of data sources, scalability, and adaptability to breeding objectives. GP models rely on the stable genetic architecture of traits and can operate independently of environmental interactions, making them highly effective for early‐stage selection and across‐year predictions (Bernardo, 2014). This stability is particularly advantageous for selecting untested lines or predicting performance in new environments where phenotypic data are unavailable. Genomic models excel at identifying additive genetic effects but may struggle to capture nonadditive interactions or environmental plasticity without Supporting Information.

In contrast, HSI provides a dynamic, environment‐specific dataset capturing phenotypic responses that directly reflect G × E interactions. By measuring spectral reflectance across hundreds of wavebands, HSI captures temporal and physiological changes in plants under varying environmental conditions. For instance, traits related to photosynthesis, water use efficiency, or heat tolerance can be derived from HSI data, which are challenging to model with genomic data alone (Krause et al., 2019). However, the utility of HSI is limited to scenarios where plants are physically present in trials, and its performance is often constrained by environmental variability, as observed in our study when predicting across years.

### 4.2 Challenges and opportunities with HSI

The challenges of HSI include managing high‐dimensional data and ensuring consistency in reflectance measurements across varying field conditions. Preprocessing steps like spectral normalization and feature reduction are critical to mitigate noise and focus on informative wavebands (Costa et al., 2021). Moreover, the limited portability of HSI data across years highlights the need for integration with genomic data, as HSI alone may not sufficiently capture the underlying genetic potential of a genotype. In this study, spectral reflectance measurements were averaged across two time points to provide a stable and streamlined dataset for integration with genomic and ECs. This approach was chosen to align with the practical constraints of large‐scale breeding programs, where simplicity and efficiency are critical. While averaging effectively captured the overall spectral signal, alternative methods such as using metrics like area under the curve or treating timepoints as separate predictors could provide complementary insights by capturing temporal dynamics more explicitly. Future studies could explore these approaches to build upon the findings of this research, potentially uncovering additional patterns related to genotype‐environment interactions and phenological changes.

GP provides a foundation for predicting long‐term genetic gains, as it is unaffected by transient environmental conditions (Crossa et al., 2017). Conversely, HSI excels in capturing short‐term phenotypic plasticity and fine‐tuning predictions under specific conditions. The two approaches are therefore complementary rather than interchangeable. For instance, in breeding programs targeting traits strongly influenced by environmental stress, combining genomic and hyperspectral data (as seen in our G + H model) significantly enhances prediction accuracy by leveraging both stable genetic effects and dynamic phenotypic responses.

### 4.3 Integration of genomic, hyperspectral, and environmental data

The comprehensive parametric model (M9) effectively integrates these data sources, modeling interactions such as G ×E and hyperspectral‐by‐environment (H × E) effects. Its superior performance in forward predictions demonstrates how combining genomic and HSI data mitigates the challenges of across‐year variability. This supports the conclusion that genomic data provide a stable backbone for predictions, while HSI refines accuracy by accounting for phenotypic variability driven by environmental interactions.

Our study evaluated the accuracy of different models in predicting wheat GY under heat‐stressed environments by integrating genomic, HSI, and environmental covariate data. HSI, while complex and data‐intensive, offers the unique ability to capture detailed spectral information across hundreds of narrowband channels, making it invaluable for predicting complex traits like GY. Combining HSI with traditional genomic SNP marker data significantly improved model precision and reliability both within and across years. Strategically, GP excels in early‐stage selection by providing stable, environment‐independent estimates, while HSI is more effective in late‐stage trials for refining predictions under specific environmental conditions. Integrating these approaches within machine learning frameworks further enhances their utility, capturing nonlinear relationships and leveraging HSI‐derived covariates to complement the genetic stability offered by SNP markers.

### 4.4 Impact of environmental variability on prediction accuracy

Year‐to‐year variations in weather had a notable impact on the relationship that was seen between GY and HSI data, with temperature, precipitation, and solar radiation dynamics shifting over the course of the years. The unique weather dynamics experienced in 2023 may have been a contributing factor to the differences in prediction accuracies that were derived from that year compared to the previous years. While the spectral heritability (*H* ^2^) for HSI data was notably lower in 2023, GY was higher than in 2021 and only marginally different from 2022 (63% vs. 62%). This discrepancy highlights the complex interplay between phenotypic heritability and environmental effects, which can influence model performance.

Additionally, the population size in 2023 was the smallest among all years, being less than half of the size of the 2021 population, further compounding the differences in predictive performance. Results from 2021‐ and 2022‐ML models show that HSI data can perform as well as or in some cases better than genomic‐based models under certain conditions. However, differing environmental conditions in each year and their corresponding influence on prediction accuracies highlight the importance of using HSI as a complement to SNP marker data rather than as a replacement, which aligns with the findings by Rutkoski et al. (2016). With the G + H (M3) approach is particularly beneficial in years where the HSI data itself have lower heritability and is less predictive.

### 4.5 Performance of ML models

Within the same year, the ML models tended to do best, this may be because these algorithms are adept at capturing complex patterns and interactions that are specific to each growing season, effectively adapting to the unique characteristics of the data from each separate year. Among the ML models, the GBR tended to outperform the rest, followed by RFR and SVMR, and lastly, ANN. The superior performances of GBR and RFR over the others may be attributed to how each algorithm methodically handles the training data. GBR is a boosting technique where the errors made by earlier trees in the training sequence are used to improve its learning. Here, each subsequent tree is made to rectify the mistakes made by their predecessors, a method that proves very efficient when handling intricate and nonlinear relationships among characteristics, often found in both genomic and hyperspectral datasets.

On the other hand, RFR constructs many decision trees and combines them to enhance its ability to generalize which can mitigate overfitting and make it resilient to noisy data, making it efficient for agricultural data prone to environmental fluctuations (Duarte‐Carvajalino et al., 2021). Contrasting with these models, SVMR and ANN may lag in this scenario as SVMR is sensitive to the choice of kernel and regularization parameters, which might not be optimally tuned for every scenario within the limited scope of a growing season (Nagasubramanian et al., 2018). ANNs, while very capable, need a substantial amount of data for good training and might suffer from overfitting if not adequately regularized or if the data provided is insufficient to handle the network's complexity (Bejani & Ghatee, 2021).

However, the ML models' rankings can change under different conditions. For example, in a scenario where data are plentiful and diverse, say from multiple locations, ANN could potentially outperform the others in terms of its ability to learn deeper representations of the dataset. Hence, when selecting a model for GP, it is important to consider not only the overall performance metrics or complexity of the model but also the unique characteristics of the dataset and the computational resources available.

On the other hand, the semiparametric models relying on RKHS explicitly include the interactions for explaining differences across environments returning better results. Like within‐year predictions, across‐year predictions that relied on HSI data alone underscored the need to incorporate genomic and HSI data. These models were outcompeted by their counterparts, which included genomic data as well as those that modeled the effects of environmental interaction. The variability of weather conditions within a given year can greatly affect the accuracy of predictions, underscoring the significance of integrating data from several sources to alleviate these impacts. Models that exclusively depend on HSI data had diminished performances all around when trained over multiple years. This is consistent with previous findings that have shown that merging several data sources frequently leads to improved prediction accuracies (Sandhu et al., 2021).

### 4.6 Forward prediction and practical breeding scenarios

With the forward prediction approach, we can get insight into patterns that emerge across years and understand how the models are able to adapt to new environments by masking a single year's data as missing values and using the remaining as the training set. Trends in predictive accuracies achieved across all the implemented models indicate that years with low prediction accuracies can largely be mitigated by incorporating data from multiple sources, other years, and shared lines across years. Our comprehensive model (M9) outperformed the others in all cases, further displaying that ECs can work in tandem with HTP data, namely, HSI, to capture the G × E.

In the forward prediction scenarios, we observed that including multiple years of data improved prediction accuracy, despite year‐to‐year environmental variability. Thus, highlighting the value of having robust historical data when predicting the performance of future lines, as also seen in other studies (Costa et al., 2021). In the analysis where the 2023 data served as the target for prediction, aligning with the breeding objective of predicting future performance based on past and present data, it is important to recognize that predicting genotypes within the same year and across years presents different challenges. Predicting across years, particularly in unobserved environments, often involves more variability in environmental and genetic interactions. The comprehensive parametric (M9) model consistently outperformed ML approaches like GBR and RFR. This outcome reflects the complexities involved with across‐year predictions where genetic and environmental interactions may vary significantly. Despite strong predictive power of ML models in specific contexts, like within a single year, across‐year predictions introduce layers of new variability that may require models to adapt differently. The across‐year prediction challenge should be viewed as complementary to the within‐year predictions, each providing unique insights into model robustness and generalization (Ang & Seng, 2021).

In addition, we also evaluated the predictive models’ ability to select the top 25% highest yielding lines (CI) in the forward prediction scenario because the target of the breeders is to identify the most promising genotypes rather than accurately predict the rankings along the complete distribution. There are little to no benefits of correctly predicting those genotypes in the middle of the population showing average performance, that is why breeders often concentrate on identifying the best genotypes rather than achieving precise rankings across the entire population (Bernardo, 2014). A correct identification of the superior individuals via the CI demonstrates the potential of the data in practical breeding scenarios. The comprehensive model was especially useful for adjusting to different environmental circumstances from one year to the next and could be extended to unfamiliar geographic areas, assisting breeders in making choices in difficult situations. So long as the environmental conditions of these unobserved environments fall within the range of those environments in the training set. The results advocate for the continued improvement of ML techniques to effectively handle environmental variations and challenges specific to certain growing areas or years, indicating that incorporating strong data‐driven strategies can contribute to the progress of resilient agricultural practices.

### 4.7 Relative performance of comprehensive and baseline models

While the comprehensive parametric model (M9) and ML models like GBR and RFR demonstrated superior predictive performance in specific scenarios, it is essential to contextualize these results relative to the simpler model (M1). The M1 model, which relies solely on genomic data, serves as an efficient baseline, offering ease of implementation and computational efficiency that is particularly attractive to breeders. However, its reliance on additive genetic effects limits its ability to account for complex interactions, such as G × E, that often play a significant role in breeding outcomes under diverse environmental conditions.

Comparatively, the comprehensive model (M9) provides substantial gains in prediction accuracy by integrating genomic, hyperspectral, and environmental covariate data while explicitly modeling interaction effects. In forward prediction scenarios, for example, M9 outperformed M1 consistently, achieving up to a 40% improvement in predictive accuracy. Similarly, the ML models, particularly GBR and RFR, showed promise in leveraging multi‐source data and capturing nonlinear interactions. However, these approaches come with added computational demands and sensitivity to data quality, making their adoption situationally dependent.

Together, our results offer a contribution to the growing body of evidence that HTP and ECs integration into GP schemes can enhance the accuracy of selections that are ultimately made. The previous studies have shown similar improvements when including spectral reflectance indices and other physiological trait data in their models (Guo, Pradhan, et al., 2020; Montesinos‐López et al., 2023). Our results show some of the potentials of advanced HTP techniques to accelerate the development of high‐yielding and heat‐stress‐tolerant wheat lines. In the future, research endeavors will put even greater focus on fine‐tuning the integration of numerous HTP sources to heighten model accuracies. By enhancing the capabilities of ML models to deal with highly dimensional data types and utilizing multi‐source data, we can expect to see robust and resilient models and broaden the scope of study to encompass larger genotyping datasets and a wider range of environmental contexts.

## 5 CONCLUSION

Genomic SNP markers, hyperspectral, and environmental data were employed in the study to improve the accuracy of predicting wheat production in environments that experience heat stress at key periods of the wheat growth cycle. The results of our study show that integrating HSI data with conventional genomic SNP markers greatly enhances the precision of GP models. Here, we demonstrate that HSI can enhance genomic data, especially during years characterized by adverse weather circumstances such as 2023. The exceptional efficacy of models that integrate different data sources and environmental factors highlights the need for a holistic approach. This integrated approach not only increases the precision of predictions but also boosts the durability and dependability of wheat breeding programs focused on creating heat‐resistant, high‐yielding lines.

## AUTHOR CONTRIBUTIONS

**Jordan McBreen**: Conceptualization; data curation; formal analysis; investigation; methodology; writing—original draft. **Md Ali Babar**: Conceptualization; funding acquisition; investigation; methodology; resources; writing—review and editing. **Diego Jarquin**: Data curation; formal analysis; software; writing—review and editing. **Yiannis Ampatzidis**: Data curation; formal analysis; investigation; resources; writing—review and editing. **Naeem Khan**: Data curation; writing—review and editing. **Sudip Kunwar**: Data curation; writing—review and editing. **Janam Prabhat Acharya**: Data curation; writing—review and editing. **Samuel Adewale**: Data curation; writing—review and editing. **Gina Brown‐Guedira**: Data curation; resources; writing—review and editing.

## CONFLICT OF INTEREST STATEMENT

The plant field trial studies in the current study adhere to the applicable institutional, national, and international rules and regulations. The necessary permissions and/or licenses were acquired for the gathering of plant or seed specimens. The authors declare that the research was conducted in the absence of any commercial or financial relationships that could be construed as a potential conflict of interest.

## Supporting information



Supplementary figures

## Data Availability

The datasets used in this study can be found at http://datadryad.org/stash/share/t8Ev6Aptra1z86ELtPNd2A0Bi1glIrwrTS3zrH4VpDg and http://datadryad.org/stash/share/UGz_RyppCD‐KCea6z0pR83oU5V2WgGzC09ADOb3kVpk.
