# Supplementary material for: Enhancing genomic‐based forward prediction accuracy in wheat by integrating UAV‐derived hyperspectral and environmental data with machine learning under heat‐stressed environments
Source: Plant Genome. 2025 Jan 8;18(1):e20554. doi: 10.1002/tpg2.20554 (PMC11711122; doi:10.1002/tpg2.20554)
Supplement: Supplementary file 1 [file TPG2-18-e20554-s001.docx]

***Supplementary material***

**Enhancing genomic-based forward prediction accuracy in wheat by integrating UAV-derived hyperspectral and environmental data with machine learning under heat-stressed environments**

Jordan McBreen^1^, Md Ali Babar^1*^, Diego Jarquin^1^, Yiannis Ampatzidis^2^, Naeem Khan^1^, Sudip Kunwar^1^, Janam Prabhat Acharya^1^, Samuel Adewale^1^, Gina Brown-Guedira^3^

^1^Department of Agronomy, 3105 McCarty Hall B, Gainesville, FL 32608, USA.

^2^Agricultural and Biological Engineering Department, Southwest Florida Research and Education Center, University of Florida, IFAS, 2685 SR 29 North, Immokalee, FL 34142, USA

^3^Plant Science Research, USDA-ARS SEA, Raleigh, NC 27695, USA.

*** Corresponding author:** [mababar@ufl.edu](mailto:mababar@ufl.edu)

Pages: 3

Supplemental Tables: 0

Supplemental Figures: 2

**Supplementary figures**

**. Scatter plots of actual vs. predicted GY for 2022 using various ML models**

Supplementary Figure S1. Model comparison for the prediction of top 25% yielding lines using different algorithms. Scatter plots illustrate the predicted versus observed yields for RFR, SVMR, and ANN models. Dashed lines denote the mean observed and predicted yield values, illustrating the central data trend. Data points are classified into quadrants A-D based on their deviation from these means: A) both predictions and observations above the means; B) both below the means; C) predictions above and observations below the mean; D) predictions below and observations above the mean.

**Bar charts of model prediction accuracy for ML models in forward prediction.**

Supplementary Figure S2. Forward prediction accuracies for GY using different ML models. Bar graphs showing Citra, Florida GY prediction accuracy (ρ) of several machine learning models. The top graphic displays 2022 growing season prediction accuracy using 2021 data. The bottom graphic shows 2023 growing season prediction accuracies using 2021 and 2022 data. Models used are Random Forest Regression (RFR), Support Vector Machine Regression (SVMR), Gradient Boosting Regression (GBR), and Artificial Neural Network (ANN). Genomic (G), Hyperspectral (H), and environmental covariate (w) data.
